# Retrospect of the Medical Sciences

**Published:** 1843-09-16

**Authors:** 


MORBID CONDITION OF THE BLOOD IN GLANDERS.

M. Renault made a number of experiments to ascertain whether the blood of an animal labouring under glanders could communicate the disease to a healthy animal. A horse was inoculated with pus taken from a man labouring under glanders; the horse was soon after seized with the disease. Before killing it a quantity of blood (ten ounces) was taken from the jugular vein, and injected into the veins of a healthy horse. At the end of three days the horse was seized with acute glanders; and blood drawn from it, and injected into the veins of another healthy and vigorous horse, in its turn produced glanders also in three days. From these and other experiments, M. Renault concludes that the blood is essentially diseased in this fearful malady.—*Prov. Med. Journ.*

### RETROSPECT OF THE MEDICAL SCIENCES.

### SUCCESSFUL CASE OF TRANSFUSION OF BLOOD.

BY J. C. PRICHARD, M. D., F.R.S., M.R.I.A.

The patient alluded to is a mercantile man, who had been in the habit of travelling in pursuit of his commercial business. He had been a strong, active man, and had lived as persons of his vocation generally do, but more temperately than many. When I saw him first, on the 11th of January, 1834, he had been upwards of two years an invalid, and had been under the care of Mr. Henry Clark, one of the surgeons of the Bristol Infirmary—a medical practitioner of great skill in this city. For some short time he had also been visited by Dr. Riley, and by these gentlemen I was called to see the patient. His early complaints had been of dyspeptic symptoms. These were followed by emaciation and loss of strength. His actual state was that of extreme inanition; his pulse was feeble, jerking, very compressible, the calibre of the artery apparently not filled; he had palpitation of the heart, increased by the slightest exertion, while any effort brought on an approach to syncope. No disease was discoverable by the sounds of the heart or respiratory organs, though some suspicion was entertained of slight dilatation.

The state of the urine alone threw some light on the nature of the disease. It had long deposited a very copious sediment, of a whitish colour, slightly tinged with purple, which was redissolved by dilution, with the addition of an alkali. It appeared to consist of lithates, with some chyle; and it was thought probable by the medical gentleman who had previously attended the patient, and by myself at our first meeting, that an exhausted and exsanguineous state, brought on by a long continued draining of the system, constituted the principal disease, or at least that which first required attention. Under these circumstances, it was determined to try a restorative and repletive plan. A nourishing diet was ordered, with malt liquor and some other stimulants. His stomach would receive but little, and at length rejected food. His exhaustion increased to that degree that immediate fatal syncope was threatened. We determined, the patient being obviously *in extremis*, to try the effect of transfusion, and as patients under cholera had borne the injection of large quantities of fluid, there seemed to be no danger in injecting a considerable quantity of blood. Sixteen ounces were taken from the veins of a hale young man, a servant of the patient, and were injected most skilfully by Mr. Clark. The patient was immediately revived and roused. On the following day he appeared much stronger, but complained of some sense of fulness about his head, and a few drops of blood escaped several times from his nostrils. This subsided; his appetite became good, and he ate plentiful meals of meat, and drank porter, &c. He gradually recovered his strength. The urine improved under the use of alkalies with lime water, and these were nearly all the remedies used except a few bottles of an effervescing solution of citrate of iron. After two or three months he left his chamber, and then his house, and I understand he is now travelling on commercial business.

I think it may be inferred from the facts of this case that many lives may be saved by injecting good blood into the veins of exhausted patients, and, perhaps, this measure may become hereafter as important a remedial measure as the detraction of blood has been since the time of Hippocrates.—*Prov. Med. Journ.*

---

### STATISTICS OF EPILEPSY.

By M. Leuret, of the Bicêtre, Paris.

The following facts are taken from the first part of an interesting paper on this subject, by M. Leuret:

Among 106 cases of epileptic patients, 24, or nearly one-fourth, began to be affected between the 10th and 14th year of their age; 18 were first attacked between their 15th and 19th year, and 16 between their 20th and 24th year; 58 patients, consequently, of the whole number were first attacked between their 14th and 24th years. Of the 106 patients, the disease was ascertained to have existed either in the father or mother in 6 instances only. And in no more than 8 cases was it found that the parents had died of any disease of the brain; there was insanity thrice; apoplexy twice; paralysis once; suicide once; meningocephalitis once. Of the 106 patients, 30 had been drunkards; 24 masturbators; and 15 addicted to women. The actual or presumed cause of the first attack was ascribed to terror, in fifteen cases; to Onanism, in twelve; to drunkenness, in six; to anger, in two; to distress, in two; to falls, in two; to libertinage, in one; &c. 30 of the 106 patients had an attack very regularly once a-fortnight; 17 suffered attacks once a-month; 13 once a-week; 9 every three or four days; 4 almost every day; 2 every day; 1 every two months; 3 every three months; and 24 at very irregular intervals. In 35, the attacks supervened in the night especially, in 29, they were as frequent in the day as in the night; in 12, they frequently occurred in the day; in 8, they occurred during the day only; in 8, in the night only; in 3 in the morning only; in 4 others, generally in the morning; and in 1, in the evening only.

*Ibid, from Archives Générales de Medecine.*

---

### THE DIAGNOSIS OF UTERINE DISEASES.

In ascertaining the diseases of the uterus, we have it in our power to avail ourselves of both the classes of symptoms of which I have spoken; or, in other words, we may form our judgment of its morbid conditions, both by studying the vital functional derangements, local, sympathetic, and constitutional, that may be present; and by informing ourselves, by physical diagnosis, of the exact existing state or states of the organ itself. Each of these classes of symptoms may afford us most important information, and in all cases where both can be had recourse to, our diagnosis will be greatly more certain under their combined evidence, than if the data furnished by either were alone trusted to. Of the two (if we are to make comparisons between them) the physical symptoms are assuredly, in most cases, by far the most valuable and trustworthy, and yet in the common course of medical practice, the testimony which they are capable of affording is but too frequently neglected and overlooked, and the functional and much less faithful class of symptoms alone relied upon, as well in forming the diagnosis as in directing the measures of treatment.

Thus, if we attempt to analyse the mode in which the medical practitioner usually endeavours, in any suspected case of uterine disease, to detect the presence and character, or ascertain the absence of such an affection, we will find, I believe, that he generally proceeds by taking into consideration some or all of the following sources and varieties of information: —

First, The local and functional state of the uterus, so far as it is indicated by the quantity, character, periodicity, &c., of the menstrual and mucuous secretion of the organ; by the occurrence or non-occurrence of morbid uterine or vaginal discharges—as of blood, serous fluid, pus, &c.; by the existence or not of morbid sensations in the region of the uterus—such as different modifications of pain, intermittent or continuous, feelings of heat, weight, tension, bearing down, &c.; and, if the patient be married, by the reproductive powers of the organ—as shown by sterility, by the recurrence of abortions, &c.

Secondly, The presence or absence of various morbid affections of the neighbouring viscera, particularly of the rectum and bladder, and of branches of the vessels and nerves passing through the pelvis, as indicative either of their sympathetic irritation or of their mechanical compression by the enlarged or displaced uterus.

Thirdly, The existence or non-existence of secondary local neuralgic pains in the mammæ, along the lower extremities, in the loins, and at points along the course of the spinal column, in the parietes of the thorax or abdomen on one or other side (and especially under the left breast, and under the margin of the ribs,) along the colon, &c., increased in their intensity by any causes of increased action in the uterus itself, by the erect posture, by menstruation, &c.

Fourthly, The state of the general constitution of the patient, as marked by various degrees of deviation from the standard of health, and especially by the supervention of nervous, hysterical, dyspeptic, chlorotic, or cachectic symptoms.

The several preceding series of morbid phenomena consist of derangements in the vital actions of the uterus, or of other parts and organs secondarily affected, or of the constitution at large, and so far strictly belong to the class of functional or dynamic symptoms only. Up to a late date in the history of uterine diagnosis, most practitioners remained, and some still remain, satisfied with the degree of knowledge which is afforded by the above sources of information. No one, however, who is practically acquainted with the disease of the uterus can have any hesitation in declaring that the symptoms derivable from these sources, are utterly inadequate, in the general routine of such cases, for the purposes of correct diagnosis, and are constantly liable to lead into fallacy and error when their individual evidence is alone trusted to. In making this observation, I do mean to allege that these classes of symptoms are not sufficient to give us the power in most, though not in all instances, of detecting the actual presence of uterine disease. They are perfectly deficient, however, in this other point, that, by their single unassisted aid, we cannot ascertain what the exact character and nature of the existing disease is, nor, consequently, what may be the proper line of treatment requisite for its alleviation or removal. They may generally, in other words, enable us to decide that the uterus is the seat of some morbid condition, but are not adequate to inform us what that morbid condition really is. They may show that the organ is affected, without showing us how it is affected.

*Prof. Simpson, in Lond. and Ed. Month. Journ.*

### CONNECTION OF CHOREA WITH RHEUMATISM—PERICARDIAL IRRITATION.

The connection of chorea with rheumatism is not the least interesting feature in its history. It often occurs in the rheumatic diathesis, as I believe is indicated by the frequent coexistence of chronic valvular disease of the heart with it, and it frequently comes on after attacks of rheumatic fever. In the case of a lad, fourteen years of age, who was in the hospital two years ago, the first attack of chorea came on four months after one of rheumatic fever; a year after this he had a second attack of chorea; some time afterwards he had another attack of rheumatic fever, and in three months another one of chorea. The records of practical medicine contain abundant evidence to warrant the assertion that *the rheumatic state of constitution is favourable to the developcment of chorea.*

"We must, however, distinguish between the true chorea occurring in rheumatic habits and those chorea-like movement which have lately attracted the attention of physicians as symptomatic of the disease of the pericardium or heart. These latter are examples of reflex movement, excited by the stimulus of irritation conveyed from the heart to the nervous centres, and thence reflected to the muscles of the extremities and the face, causing in the latter a peculiar sardonic expression, which to the practised eye is very characteristic of pericardial irritation."

*Dr. Todd's Clinical Lecture in Med. Gaz.*

### NEW TEST FOR ARSENIC.

The one-hundred-and-forty-fourth part of a grain of arsenious acid was mixed with two fluid drachms of milk. The mixture was boiled with a few drops of muriatic acid, and a slip of copper was introduced. In less than a minute the metal was coated with a grey film of metallic arsenic. Several pieces were thus coated; they were washed in water, dried in the heated current of air over a spirit lamp-flame, and introduced into a small reduction tube. On applying a gentle heat to the copper, octohedral crystals were obtained; visible to the eye in the light of the sun, but plainly distinguishable with a lens of low power. The crystals dissolved in water gave the usual reactions with the ammonio-nitrate of silver and sulphuretted hydrogen gas. In a second experiment, the same quantity of arsenic was mixed with two drachms of porter; in a third, a like quantity with two drachms of gruel, and the copper test was applied with similar satisfactory results. Each experiment was completed in about five minutes. Brandy, containing a poisonous impregnation of arsenic, was then tested; and the arsenic readily separated. A few drops taken from a bottle of port wine, containing arsenic, and which had nearly caused the death of three persons about four years ago, were next submitted to the test, and the metal readily obtained. In organic solids the results were equally striking: a few grains of a cake containing arsenic, which had been used in an attempt to poison, were boiled in a small quantity of distilled water, and muriatic acid and copper added, when metallic arsenic was immediately precipitated. The contents of the stomach of a person who was poisoned by arsenic in February, 1834, which had been loosely exposed, and allowed to become decomposed during a period of upwards of nine years, were next examined. A few drops of the thick turbid liquid were placed in a tube and boiled with dilute muriatic acid and water; the copper introduced was covered with a bright layer of metallic arsenic. The contents of three other stomachs taken from persons poisoned by arsenic in 1835, 1838, and 1840, gave precisely similar results. In the last case, the whole of the contents, with the food at the time contained in the organ, had been evaporated to a dry solid mass; a few grains of this were sufficient to furnish a clear demonstration of the presence of arsenic by the aid of the test. In the conversion of the metal to arsenious acid, it will at once suggest itself that if octahedral crystals should not be obtained by heating one portion of copper, several slips should be introduced together or separately. In all these cases arsenic will have been discovered by the application of Marsh's test, or sulphuretted hydrogen gas; but the process would have occupied a much longer time, and with regard to Marsh's test the metallic arsenic could not have been so speedily converted to arsenious acid in a form convenient for the identification of its properties.

Another useful application of the copper test may be made in the following way. If the arsenic have been thrown down from an organic liquid in the form of impure sesquisulphuret, this may be dried and deflagrated with nitre, or decomposed by nitro-muriatic acid, whereby arseniate of potash or arsenic acid will result, and the organic matter will be entirely decomposed. In the case of deflagration by nitre, the surplus nitric acid should be expelled by sulphuric acid, and the arseniate dissolved out of the residue; or if nitro-muriatic acid be used, the liquid may be evaporated to dryness. On boiling either of these products with copper and muriatic acid, the metallic arsenic will be readily procured.—*Mr. Taylor, in British and Foreign Review.*

### EMPLASTRUM CERATI SAPONIS.

Although this plaster has for many years been extensively sold, spread on linen or calico, we have not seen any published formula for it, and the soap cerate being too soft for use as a plaster, several correspondents have inquired how they should proceed when emplastrum cerati saponis is ordered in a prescription.

The addition of one part of ceratum saponis to two parts of emplastrum plumbi, forms a plaster which, on an emergency might be used in such a case, the article in question not being a preparation of the Pharmacopœia. But the most legitimate mode of reducing the cerate to the form of a plaster is to continue the evaporation until all the vinegar is expelled. This method is adopted at the Army Laboratory, where the plaster is kept, not only spread as usual, but also in rolls like other plasters.

The following is the formula used at the Army Laboratory:—

| | |
|---|---|
| Common vinegar (No. 24) old measure. | 8 gallons, |
| White Castile soap, | 16lb. |
| Yellow wax, | 20lb. |
| Olive oil, | 32lb. |
| Litharge, | 32lb. |

Boil the litharge with the vinegar almost to dryness; remove from the fire and add the soap, previously scraped or cut; replace on the fire, taking care to avoid empyreuma; then add the wax and oil, melted and strained, and continue the evaporation, stirring constantly until the whole of the vinegar is expelled. The time required for completing the above quantity is three or four days.

The original recipe directed eight gallons of the residuum of distilled vinegar to be used instead of twenty-four gallons of common vinegar. The plaster thus prepared has a dark-brown colour; but when the vinegar is used and the plaster has been carefully made, its colour is not much darker than that of adhesive plaster. The emplastrum cerati saponis extensum, as usually sold, is coloured artificially with burnt sugar.—*Pharmaceutical Journal.*

### CASE IN WHICH A FOREIGN BODY WAS LODGED IN THE RIGHT BRONCHUS.

BY SIR BENJAMIN B. C. BRODIE.

The author's object in this paper was to describe a case in which a half-sovereign was lodged in the right bronchus of the patient for a period of thirty days, and in which certain novel measures adopted for its removal proved successful. It was on the 3d of April, while the patient, Mr. B., was amusing some children, that the coin which he had in his mouth accidentally slipped into the trachea. The symptoms which succeeded were principally occasional severe fits of coughing, and a sense of pain referred to a part of the chest corresponding to the situation of the right bronchus. No particular sounds were detected by the use of the stethoscope. The patient was able to pursue his usual avocations, and made two journies into the country. On the 19th of April, having placed himself in the prone position, with the sternum resting on a chair, and his head and neck inclined downwards, the patient had a distinct perception of a loose body slipping forwards along the trachea; a violent convulsive cough ensued, and, on resuming the erect posture, he again had the sensation of a loose body moving in the trachea towards the chest. An apparatus of the following kind was now constructed. A platform on which the patient could lie prone, was made to move on a hinge in the centre; so that one end of it being elevated the other was equally depressed. On the 25th April the patient was laid on this apparatus, with his shoulders and body fixed by means of a belt, and his head was lowered to an angle of nearly ninety degrees with the horizon. His back was then struck several times with the hand, but violent fits of choking were brought on each time, and it was not deemed prudent to continue the experiments. On the 27th it was agreed in consultation to make an opening in the trachea, between the thyroid gland and the sternum. In proposing this, the object was twofold: 1st, that an attempt might be made to extricate the coin by the forceps; 2d, that if relief could not be obtained in this manner, the artificial opening might answer the purpose of a safety-valve, and the experiment of inverting the body on the platform be repeated without the risk of causing suffocation. The operation having been performed, several attempts to extricate the coin were made, but without success; and, on each introduction of the forceps, paroxysms of convulsive coughing of such a violent kind were brought on, that it was plain that the attempts could not be persevered in without danger to life. On the 2d of May, a renewal of those trials was followed by the same results. On the 13th, the wound in the trachea having been kept from closing by the occasional introduction of a probe, the patient was placed on the moveable platform as described before; his back was then struck by the hand; two or three efforts to cough followed, and presently the patient felt the coin quit the chest, striking almost immediately afterwards against the incisor teeth of the upper jaw, and then dropping out of the mouth. No spasm of the muscles of the glottis took place; a small quantity of blood was ejected at the same time, apparently coming from the granulations of the external wound. From this date the patient proceeded rapidly to get well.

The author concluded by making observations on the following heads:—1st, on the influence of the size, weight, and form of a foreign body introduced into the windpipe, in modifying the symptoms; 2d, he referred to experiments which showed that a heavy body, like the coin in the present case, was most likely to drop into the right bronchus; 3d, he adverted to the want of success attending the use of the stethoscope in this and in some other cases of the same kind; 4th, he pointed out the reasons on which he had founded his opinion, that the artificial opening made in the trachea would prevent spasm in the glottis, and thereby give greater chance of success to the experiment of inverting the patient's body on the moveable platform; lastly, he dwelt on the difficulties and dangers attending the use of the forceps, when a weighty body is lodged deeply in one of the bronchi, as was the case in his patient.—*Trans. Royal Med. & Chirurg. Soc. from Lond. Med. Gaz.*

---

### INFLUENCE OF RICKETS UPON THE GROWTH OF THE SKULL.

BY ALEXANDER SHAW, M.D.
Surgeon to the Middlesex Hospital.

This paper was the sequel of one by the same author, printed in the 17th vol. of the Transactions of the Royal Medical and Chirurgical Society. The object of the former publication was, in the first place, to prove that, besides causing softening and distortion of the osseous system, rickets has the effect of arresting the process of growth; and secondly, to show that, owing to this interruption, the proportions of the figure peculiar to the adult are not perfectly attained by persons affected with the disease, but continue to be more or less those of the child. The figure of the child is characterised by the head, trunk, and upper extremities being of large dimensions compared with the pelvis and lower extremities, while that of the adult has the former parts relatively small, and the pelvis and legs large and powerful. In persons deformed from rickets, the whole figure is stunted; but the head, trunk, and upper extremities together, when compared with the natural adult dimensions, are only defective to a slight degree, (one-thirteenth,) while the pelvis and lower extremities are defective to a great degree, (one-third.) This difference the author accounted for by suposing that, as the disease stops the growth, it interrupts at the same time the change then in progress of being produced in the relative proportions of the figure; and so causes the patient, when arrived at adolescence, to exhibit traces of the configuration of the child. Having referred to the importance of this view in relation to the size of the pelvis in child-bearing women deformed from rickets, and stated that by measuring numerous specimens he had ascertained that the defect of growth in this part amounts, on an average, to nearly a quarter of the natural size, he proceeded to apply the same principle to the explanation of a peculiarity in the form of the head which he had observed as a general character in ricketty persons. This peculiarity consists in a disproportion between the size of the cranium and of the face. Between infancy and adolescence, a change in the relative proportions, analogous to that which occurs in the figure generally, takes place in the skull. Near birth, its form is characterised by the cranium being of a large bulk compared with the face: while at adolescence the contrast is greatly diminished, owing to the face having become much bulkier in comparison with the cranium than it had originally been. The author had remarked, and confirmed his observations by numerous measurements, that the skull, in ricketty persons, does not attain the proper adult proportions; but, on the contrary, the cranium appears remarkably large compared with the face, just as during childhood. Thus, taking the dimensions of the face as the limit of comparison, he found that in the skull of the infant the size of the cranium is as 8 to 1; in the adult as 6 to 1; and in the ricketty person (although beneath the standard size) as 7 to 1. He explained this disproportion by supposing that, as rickets arrests the growth, it interrupts, at the same time, the change occurring in the relative proportions of the skull between infancy and adolescence; and thus gives rise to the childlike character of the proportions. The proposition was illustrated by showing the contrary effects which an increased activity of the growth produces. In the figure generally, when the growth has been preternaturally active, as in tall persons, the effect of the unequal rate of developement in the two divisions of the frame is shown by the lower extremities acquiring an undue length compared with the trunk; so, in the head, the face becomes disproportionately larger compared with the cranium: thus by measuring the skull of the giant preserved in the museum of the College of Surgeons, the author found that the dimensions of the cranium (although above the standard size) are, to those of the face, only in the proportion of 5 to 1 in this skull. Having next shown that the orbits always preserve a uniform size, whatever be the dimensions of the face, in skulls of different proportions, and accounted for this fact by referring to the anatomical relation of the frontal and maxillary sinuses to these cavities, and showing that the two sinuses vary in capacity according to the rate of growth, he passed to the consideration of the growth of the maxillary bones. After dwelling on the difference in the mode of formation of the teeth as compared with the jaw bones, and the importance of an exact relation being preserved between the development of both parts, and referring to the observation of Hunter regarding the different rates of growth in the anterior and posterior divisions of the maxillary bones, he concluded by showing that, as rickets has the effect of interrupting the growth of the jaw bones, it deranges also the process of evolution of the teeth. *Lond. Med. Gaz.*

---

### MEDICINAL USE OF SAFFRON.

In several cases of obstinate chlorosis, that had not yielded to preparations of iron, in one case of puerperal fever, in which digitalis and bleeding had failed, and in two cases of chronic artero-phlebitis, Dr. Morgante, of Verona, reports that he has employed saffron with the greatest success, commencing with doses, in the form of pills, amounting to sixteen grains in the twenty-four hours, increasing the doses until the quantity is doubled. As to the manner in which the medicine acts—it is reported to be particularly effective in cases of increased action of the capillary vessels, and analogous in its effects to the more active preparations of iron.—*Ibid, from Mem. della Med. Con.*

### DR. PERCY ON DIABETES MELLITUS.

1. Although the liquid of the stomach is capable of inducing the conversion of starch into sugar by digestion at a suitable temperature, yet we do not derive satisfactory and demonstrative evidence that this conversion takes place in a sensible degree in the stomach in the condition of health, and under ordinary circumstances.

2. As the formation of dextrine is the first step in the conversion of starch into sugar, it is required to ascertain whether dextrine may not be the ordinary product of the digestion of amylaceous matter, in the condition of health, and whether it may not be directly absorbed.

3. In diabetes mellitus sugar is found in the stomach, the intestines, the blood, and some other secretions.

4. In this disease, also, sugar is reported to be found in the stomach, even after the exclusion of all vegetable matter from the food. On this point further observations are desirable.

5. It is asserted that the quantity of sugar in the urine, *cæteris paribus*, varies directly with the quantity of amylaceous matters in the food. On this point, also, future observations may throw much light.

6. Sugar is certainly formed in the stomach in diabetes.

7. It is not clearly ascertained whether previously assimilated matter may not be absorbed, and be capable of conversion, in a partial degree at least, into saccharine matter.

8. The disease may exist independently of any appreciable disorganization of structure whatever.

9. Enlarged mesenteric glands, and the deposition of tubercular matter in the lungs, are, perhaps, the most frequent structural changes in diabetes. Enlargement, and a flaccid condition of the kidneys, cannot be regarded as indicating alteration of structure.

10. The kidneys appear simply to eliminate the sugar from the blood.

11. Our knowledge of the state of the blood and the secretions in diabetes is yet very imperfect, and has hitherto shed no light upon the pathology of the disease.

12. Disturbance or suppression of the function of the skin almost constantly exists.

13. The salivary secretion is generally much diminished, and has an acid reaction; the reaction of saliva in health being feebly alkaline.

14. A saccharine state of the urine may exist without the actual symptoms of diabetes.

15. Sugar, according to Dr. Prout, is not unfrequently present in the urine of gouty and dyspeptic persons; and, so far as the experience of that distinguished physician has hitherto extended, invariably exists in the urine when the system is affected with carbuncle or malignant boils.

16. Thirst constantly occurs in connection with confirmed diabetes.

17. The appetite is sometimes inordinate, at other times capricious and much diminished.—*Lond. Med. Gazette.*

### AGENCIES INFLUENCING THE CIRCULATION AND CONDITION OF THE BLOOD IN REPTILES.

The researches of Gluge on the circulation in frogs have established the following facts:—On the sudden immersion of the feet of a frog in hot water the circulation is at once arrested and the blood coagulated; but the progress of the blood is soon re-established in the toes, and gradually in the other parts of the foot. The circulation (in frogs) is kept up for about twenty-five hours after the destruction of the brain and spinal cord. After the section of the crural plexus the same process is maintained for upwards of six days in the paralysed extremity, and it lasts ten days when only the spinal cord is cut at some point between the heart and the lower extremities. A considerable loss of blood greatly disturbs the circulatory process, paralyses the heart's action, and quickly destroys nervous excitability. The elliptical globules in the blood also diminish greatly in number, and are replaced by spherical globules. On suppressing the transpiration, as by varnishing the surface of the body, the blood remains liquid, and the cellular tissue and serous membranes become filled with fluid. When the respiration as well as the transpiration is suppressed, as in the case of frogs plunged into oil, the circulation is still kept up for two days.

A frog was inoculated with the blood of a man attacked with farcy; in three other frogs shreds from the stomach of a putrefying corpse were inserted beneath the skin; and under the skin of a fifth reptile a portion of decomposing muscle was placed. Paralysis of the heart and interruption of respiration ensued, with an altered state of the blood and exudation of it through the tissues,—in fact, true scurvy. No immediate effect was perceptible on the nervous system, but this soon became secondarily affected consequently on the alteration of the fluids.—*Lancet, from Schmidt's Jahrbuch.*, vol. xxxvi.

### DIAGNOSIS OF PARAPLEGIA.

When paraplegia originates in disease of the spinal cord itself, retention of urine or irritability of the bladder often announces the approach of the disease long before the loss of power in the limbs becomes evident; whereas, in all those cases in which the paralysis creeps from the extremities along the nerves towards the spinal marrow, the bladder is affected only at a late period of the disease.—*Dr. Graves' System of Clinical Surgery.*

A Russian chemist has found out a method of ensuring a supply of milk extemporaneously. He evaporates newly-drawn milk at a gentle heat till it is converted into a fine powder, which is kept in closely stopped bottles, and affords good milk on being mixed with water, even after a considerable lapse of time.—*Lond. Lancet.*

### VOYAGE UP THE MIDITERRANEAN IN THE TREATMENT OF PHTHISIS.

Statistical facts show that pulmonary complaints are more fatal amongst our troops serving at home than in the Mediterranean; and all the circumstances, independent of climate, so far as I am acquainted with them, affecting the question appear to be in favour of the troops serving at home, especially the cavalry; I am not only not able to adopt the opinion referred to, that the climate of the Mediterranean is more productive of disease of the chest than our own climate, but am obliged to fall back on the old and hitherto generally received opinion of an opposite nature.—*Dr. Davy.*

### OXALIC ACID.

Mr. Orfila states that "the urine of dogs, subjected to the action of oxalic acid diluted with water, ordinarily deposits insoluble oxalate of lime, which is not the case with that of the same animals in a healthy state, unless they have eaten sorrel."—*Prov. Med. Journ.*

### CAUSE OF BRUIT DE SOUFFLET IN CASES OF ANÆMIA.

According to M. Andral, the causes capable of giving rise to the anæmial bruit are reducible to the following:—

First, when the globules have diminished sufficiently to be below the cipher 80, the bruit de soufflet exists in the arteries in a constant manner. I have never found a single exception to this law.

Second, when the globules have remained above the cipher of 80, the bruit de soufflet can again show itself, but it is no longer constant; we continue to hear it often enough when the cipher of the globules oscillates between 80 and 100; it is met with again, but much seldomer, according as the cipher of the globules passes 100, and finally, it is no longer observed when the cipher of the globules is raised above the physiological mean.

Moreover, whatever is otherwise the nature of the disease in which these diminutions of the globules exist, the bruit de soufflet of the carotids does not show itself the less; I have proved its existence in the most different cases, putrid fevers, eruptive fevers, pneumonias, acute articular rheumatism, and in a great number of chronic diseases. But in all these cases it only took place with the ciphers of the globules marked above.

The bruit de soufflet is often present in women with child, which is in relation with the frequent diminution which the globules undergoes in them.

*Ibid from Andral's Hématologie.*

### PHYSICAL CONDITION OF THE BLOOD IN PLETHORA.

The physical properties of this blood are perfectly explained by the nature of the changes which its composition has undergone. Thus, before coagulation, the blood in plethora is remarkable for its deep coloring, which is referable to the great proportion of the globules which it contains. When it is examined after coagulation, the serum is generally observed to be more or less colored, the clot is large, voluminous, of moderate firmness, and contains much serum; a buffy coat is never found on its surface; at most we are able to observe occasionally a thin and transparent pellicle, or some scattered rays, if the blood have flowed very rapidly from the vein. The considerable size of the clot manifestly depends on the great number of the globules; and its softness, as well as the constant absence of the buff, depends on the weak proportion of the fibrine relatively to that of the globules.

The superabundance of the red globules coincides in them with a certain modification of the physiological state, and thus with a certain number of pathological facts which appear to be a consequence of it. Thus, all the functions are generally more active, and there is a superabundance of life; digestion is quickly performed; respiration is favoured by the great developement of the thoracic cavity; the circulation is rapid; the heart beats with force; but it is wrong to admit, as he has done, that in like cases its beatings may be accompanied with *bruit de soufflet.* I have advanced this opinion myself, but more attentive and longer observation has convinced me that it was never so, and that, consequently, in cases where a *bruit de soufflet* in the heart or arteries has been observed in plethoric people, it was because the diagnosis was badly founded, and because, besides the plethora, there was another disease.

Individuals whose blood contains a superabundance of red globules are subject to some special accidents, of which hitherto a very satisfactory explanation has been given. Thus the vertigos, dimness of sight, noise in the ears, heat in the head, which they experience, have been explained by congestions of the blood to the brain; but these congestions have never been, in similar circumstances, anatomically stated, and the passage alone of a superabundant quantity of globules across the vessels of the encephalon appears to me a circumstance sufficient to account for it; but what is strange is, that if it happens, on the other hand, that globules in too small number traverse these small vessels, analogous accidents will yet present themselves, so that a quantity of globules, either too great or too small, trouble certain cerebral acts in the same manner.

*Ibid, from Ibid.*

### PROGNOSIS IN CASES OF UTERINE HÆMORRHAGE.

Where recovery is to take place after uterine hæmorrhage, says Dr. M. Hall, the pallor of the countenance, the disposition to syncope, the coldness of the extremities, the feeble state of the pulse, and interrupted respiration, pass gradually away. Where the case is to terminate fatally, the symptoms gradually assume a more alarming aspect, the countenance becomes pale and sunk, the respiration stertorous, and the pulse cannot be felt at the wrist. There is great restlessness, and before death one or more fits of convulsions sometimes occur. Where recovery takes place in some women, it is astonishing how little permanent inconvenience is felt from the great loss of blood which they have sustained. In the course of ten days or a fortnight the effects have entirely disappeared; and this is the most common result. In some women, a violent determination of blood takes place to the brain, marked by heat, strong pulsation of the carotid and temporal arteries, intolerance of light, and all the symptoms of inflammation of the brain or its membranes. A strong febrile attack is also sometimes experienced without an increased determination of blood to any particular organ. These affections of the brain and nervous system are aggravated by depletion. The patient should be kept in a cool, dark room, and mild cathartics, anodynes, and antispasmodics, occasionally given. Where there is much headache and throbbing, a few leeches should be applied to the temples, and a cold lotion to the scalp.—*Dr. Lee, in Prov. Med. Journ.*

### STOMATITIS ULCEROSA.

Under this name Dr. Stober describes an ulceration of the inner surfaces of the cheeks and the edge of the tongue, which is deep and uneven, and have edges which bleed easily, and a dirty greyish-yellow bottom. It attacks only one side of the mouth, is oblong, and exactly corresponds with the dental arch, so that when the mouth is shut the ulcerated parts touch the teeth. The teeth are covered with tartar; the breath is fœtid, and the glands on the affected side of the neck are swollen. The author believes incrustation or caries of the teeth to be the cause of the disease.—*Med. Gaz.*

